# Top orthopedic sports medicine procedures

**DOI:** 10.1186/s13018-018-0889-8

**Published:** 2018-07-31

**Authors:** Sebastiano Vasta, Rocco Papalia, Erika Albo, Nicola Maffulli, Vincenzo Denaro

**Affiliations:** 1 Department of Orthopaedic and Trauma Surgery, Campus Bio-Medico, University of Rome, Via Alvaro del Portillo, 200 Rome, Italy; 2 Department of Musculoskeletal Disorders, University of Salerno School of Medicine, Salerno, Italy; 3 Centre for Sports and Exercise Medicine, Barts and The London School of Medicine and Dentistry, Mile End Hospital, London, UK

**Keywords:** Orthopedic sports injury, Anterior cruciate ligament, Posterior cruciate ligament, Meniscus, Rotator cuff, Shoulder instability, Ankle instability

## Abstract

Orthopedic sports medicine is a subspecialty of Orthopedics that focuses on managing pathological conditions of the musculoskeletal system arising from sports practice. When dealing with athletes, timing is the most difficult issue to face. Typically, athletes aim to return to play as soon as possible and at the pre-injury level. This means that management should be optimized to combine the need for prompt return to sport and to the biologic healing time of the musculo-skeletal. This poses a great challenge to sport medicine surgeons, who need to follow with attention to the latest scientific evidence to offer their patients the best available treatment options. We briefly review the most commonly performed orthopedic sports medicine procedures, outlining the presently available scientific evidence on their indications and outcomes.

## Background

About 11 athletes out of 100 participating in the Olympic Games will face an injury, based on the data from the last three editions [1–3]. Although the highest incidence occurs in contact sports, such as football, American football, hockey, and martial arts, some of the non-contact sports as athletics are equally affected by high rates of injuries [1]. Looking again at the Olympic Games data, about half of the injuries involves the lower limb, 20% the upper limb, while head, neck, and trunk account all together for 25% [1]. About a quarter of all the injuries are overuse injuries, which are more likely to occur in non-contact sports. Regardless of whether an injury is acute or overuse, the main issue in dealing with athletes is timing. The deeply engrained strong desire to return to play, within the shortest time, is the main challenge for sport medicine surgeons and drives treatment choices.

Here, we review the most commonly performed sports medicine procedures, looking at the latest evidences on the indications and outcomes.

### Lower limb

#### Knee

##### Meniscal injuries

Although meniscal injuries are mainly encountered in athletes involved in pivoting maneuvers [4], even low-impact sports such as swimming have been plagued by meniscal lesions [5]. Meniscal injuries are one of the most common musculo-skeletal issues and one of the most common orthopedic surgery performed worldwide. Vertical peripheral longitudinal tears [6–8] as well as root tears [9] should be repaired, leading to superior outcomes in terms of symptoms, function, return to play, and cartilage preservation compared to meniscectomy. In more recent years, there is an increasing body of evidence in favor of repairing horizontal tears, especially in the young patient [10]. Return to sports after meniscus repair for high-level athletes (basketball, American football, baseball) varies between 80 and 90% [11]. However, studies on meniscal repair in athletes have found that up to one third of the patients underwent reoperation for pain [12–16]. Concerning the surgical technique of a meniscal repair, there is general agreement to start the procedure performing a debridement and abrasion of the meniscal lesion walls to favor local bleeding [17], and with regard to the suture technique, vertical or horizontal sutures are recommended [18, 19], performed either with all-inside, inside-out or outside-in techniques, since no superiority have been demonstrated of one technique over the others [20, 21]. However, it has been demonstrated that vertical suturing configuration has superior load to failure values compared to a horizontal configuration [22]. Usually, all-inside sutures are used on the far posterior segments, and inside-out for the middle and anterior meniscal segments. Alvarez-Diaz et al., in a case-series on 29 competitive football players, reported that 26 (89.6%) were able to return to play at the same level of competition at a mean of 4.3 months after surgery [23].

##### Anterior cruciate ligament injury

Anterior cruciate ligament (ACL) tears, accounting for about 200,000 injuries per year in the USA, may compromise knee stability and affect negatively sports activity. Although most of the ACL tears result from a non-contact injury, lesions during contrasts in sports such as American football, rugby, or hockey are frequent as well.

Many risk factors have been identified for ACL tear. Some depend on bone morphology (narrower intercondylar notch widths, smaller notch width index, and increased tibial slopes [24]), some others on hormones and gender [25]. Recently, biomechanical factors such as limited hip internal rotation have been associated to ACL tear injury [26]. Conservative management of ACL tears can produce acceptable results, but in athletes, surgical reconstruction is usually preferred [27]. The main advantage of ACL reconstruction (ACLR) is to restore knee stability what will eventually help to prevent secondary injury to menisci and articular cartilage [28]. Different techniques are used to perform ACLR. Transtibial independent drilling (through the antero-medial portal) and the outside-in techniques are the most commonly used ones. A systematic review showed that independent drilling is more likely to lead to better biomechanical and functional outcomes compared to transtibial technique [29], although no clear clinical evidences support its superiority [30]. Outside-in drilling has the advantages of unconstrained tunnels placement, but it needs two incisions [31]. Single- or double-bundle graft reconstructions have been proposed. Biomechanical studies favored double-bundle graft in terms of rotational stability [32]; however, no clinical superiority has been shown [33].

Concerning the graft, it is possible to identify three main categories: autograft, allograft, and synthetic graft. Synthetic graft showed high failure rate, although newer synthetic grafts seem, in the short term, more promising than the older ones [34].

Allografts are expensive; there is a risk for infections transmission, delayed incorporation, and late failure [35, 36]. Therefore, especially for young athletic individuals, the choice falls on autografts: bone patellar tendon bone (BPTB), hamstrings, and less commonly quadriceps tendon. There is a huge debate on what is better between patellar tendon and hamstring, and no definitive superiority has been shown [37]. BPTB grafts ensure greater post-operative knee stability but exhibit a higher complication rate [38].On the contrary, hamstring autograft, for which revision surgery is most frequently required, is often associated with post-operative antero-posterior knee laxity, particularly in females [39]. However, despite all the great efforts trying to improve ACL reconstruction, the rate of patients who return to sports ranges between 63 and 92% [40, 41]. This trend confirms the importance of psychological factor and neuromuscular focused rehabilitation as essential elements in determining the return to play rate [42].

##### Posterior cruciate ligament injury

Isolate posterior cruciate ligament (PCL) injury has an incidence that ranges between 3 and 44% after acute trauma to the knee [43]. A PCL injury is often part of complex knee injuries, associated respectively in 46, 31, and 62% of cases, to ACL tears, medial collateral ligament (MCL) injury, and posterolateral corner injuries [44]. Isolated PCL injuries in the knees with reduced joint laxity and absence of other peripheral lesion are generally managed conservatively even in athletes, with satisfactory subjective results, and return to sport at the same level in about 50% of cases [45]. In patients with PCL injuries managed conservatively, 41% of the subjects at 14-year follow-up develop early osteoarthritis, with progressive reduction of joint function [46]. Surgical treatment can be performed to optimize joint function. After surgery, competitive sports are practiced again by 67% of the subjects and high functional demand sports by 26% [47]. Clinical outcomes show no differences between single- and double-bundle reconstruction techniques at approximately 30 months of follow-up. However, double-bundle PCL reconstruction mainly improves objective measurement of knee stability [48, 49].

#### Hip

##### Femoroacetabular impingement

Femoroacetabular impingement (FAI) is a common cause of hip and groin pain in young active people and athletes. FAI initially causes chondral lesions and labral tears and, subsequently, early arthritis [50, 51]. Arthroscopic surgery is undertaken in subjects refractory to conservative management, involving targeted physiotherapy and oral anti-inflammatory drugs. Few studies reported data about surgical procedures, such as femoroplasty, acetabuloplasty, and labral reconstruction, in athletes, and they showed that arthroscopic surgery is effective in terms of both clinical and functional improvement and return to sport (87% of patients) [52]. Moreover, timing for arthroscopic treatment is essential; the length of athletic career was significantly affected by symptom duration before arthroscopic treatment in professional hockey players [53].

#### Achilles tendon

Achilles tendinopathy is common in athletes, accounting for 6–17% of all injuries in running athletes [54–56]. The etiology of tendinopaty is still not well understood, but multiple factors play a role in its pathophysiology [57]. Pharmacological interventions currently lack for scientific evidences supporting their use [58], while conservative management of chronic midportion Achilles tendinopathy by eccentric exercises and extracorporeal shock waves therapy (ESWT) is strongly supported by several level I studies [59]. Patients resistant to those conservative measures can undergo to high-volume image-guided injection, effective in relieving patients’ symptoms and restore tendon function both in the short and long term [60]. In non-insertional Achilles tendinopathy, minimally invasive multiple percutaneous longitudinal tenotomies are valid alternatives to more invasive procedures, at least in patients without evidence of paratendinopathy, unresponsive to 6 months of conservative management. This method leads to satisfactory outcomes in the long term and restores ankle function [61]. Given the renowned complications in terms of infections and difficult wound healing, in most recent years, tendoscopy has been proposed, with satisfactory outcomes, as an alternative method to open surgery, including debridement alone or debridement associated to flexor halluces longus tendon transfer [62].

Eccentric exercises for insertional Achilles tendinopaty did not show excellent outcomes, while ESWT did better according to a quite recent systematic review [63]. For those patients not responding to conservative measures, surgery is indicated in order to debride the tendinopathic tissue and/or to excise the calcaneal bony prominence, with or without tendon detachment and subsequent fixation to calcaneus tuberosity. Surgical management of insertional Achilles tendinopathy is indicated after failure of 3- to 6-month period of nonsurgical management. Thorough debridement is necessary, and this leads often to a near to complete detachment of the tendon’s insertion. Therefore, repair with two anchors is recommended; indeed, it is associated with good to excellent results [64]. Outcomes from surgery can be satisfactory, but prolonged rehabilitation is necessary [63, 65, 66].

#### Ankle

##### Lateral ankle ligaments

Ankle sprains are common, especially in team sports [67]. They account for up to 40% of all athletic injuries, and 29% of American football injuries can be attributed to ankle injuries [68]. The most common pattern of injury is forefoot adduction, hindfoot inversion, with the tibia externally rotated and the ankle in plantar flexion. This mechanism leads to tears to one or more of the lateral ligaments of the ankle [69]. Up to 70% of the sprains involve the anterior talo-fibular ligament (ATFL) alone [67]. More than 50% of the ankle sprains do not come to medical attention [70]. Patients with ankle sprain grade I and II, accounting for greatest part, will benefit from the use of RICE (rest, ice [cryotherapy], compression, and elevation) [71]. Grade III has a less standardized management. Some authors have proposed surgical repair for the acute grade III lesion [72], but many others have reported discouraging outcomes following acute repair in favor of functional treatment [73, 74].

Chronic ankle instability is defined as the condition of symptomatic ankle instability following an acute ankle sprain managed with conservative measure [75].

Surgical management of the lateral ligaments of the ankle can be performed with an anatomic reconstruction (the Brostrom-Gould technique) or by several techniques in which a tendon graft, either autograft or allograft, reinforces the local tissue [75]. A recent comparison of lateral anatomic ankle repair (Brostrom-Gould) to allograft reconstruction showed that no revision was needed in patients from both groups, and no significant differences between groups in terms of function and patients’ satisfaction were found [76].

Allografts showed good to excellent results in up to 85% of cases, and they should be considered when multiple ligaments are involved, such as calcaneofibular ligament (CFL) other than ATFL [77].

At the time of surgical repair of ankle lateral ligaments, it is highly recommended to perform an ankle arthroscopy, since this will allow looking for intrarticular lesions [78].

Recently, different minimally invasive techniques have been proposed for chronic ankle instability (arthroscopic repair, non-arthroscopic minimally invasive repair, arthroscopic reconstruction, and non-arthroscopic minimally invasive reconstruction). However, there is still a lack for high-level evidences to support their use in daily clinical practice [79].

##### Ankle osteochondral lesions

About 50% of acute ankle sprain leads to chondral lesions of the talus (OCL), causing persisting pain despite conservative or surgical treatment [80]. Most of the OCL are central-lateral (49%) and follow an inversion ankle sprain [81]. Those kinds of lesions respond poorly to conservative measures. In the acute setting, arthroscopic debridement with excision of the loose OCL is usually indicated for lesions smaller than 1 cm^2^ [82]. Fragment fixation using bioabsorbable screws is advised for wider lesions [83].

Chronic OCLs are classified in five types according to Loomer et al. [84]. Type I and II lesions can be addressed by retrograde drilling, a technique that aims to bone marrow stimulation [85]. Type III and IV lesions can be addressed with satisfactory outcomes either by excision or curettage [86]. Finally, type V, a cystic lesion, is usually bone grafted or filled using osteochondral plugs (mosaicplasty) [87].

### Spine

#### Disc herniation and disc degeneration disease

Injuries of the cervical spine frequently affect athletes who practice contact sports and represent 44.7% of all American football-related spinal injuries [88]. In patients with diagnosis of cervical disc herniations, surgical indications are persistent symptoms, evidence of spinal cord compression on MRI and cervical myelopathy. However, drug (such as anti-inflammatory medications) and physical therapy (strengthening exercises) remains the first-line treatment and produces a higher rate of return to sport if compared with surgical treatment. Furthermore, contact sports are contraindicated in patients who underwent multilevel anterior cervical discectomy and fusion (ACDF) or with cervical myelopathy, residual pain, or muscle weakness [89, 90].

Low back pain, reported by 75% of elite athletes, is frequent in athletes of all levels [91]. In addition to genetic and anatomic factors, the incidence of lumbar disc degeneration is justified by an abnormal and increased load on the lumbar spine arising from sports activity practiced over the years, which, in the long term, leads to early disc degeneration [92]. Similar to the upper spine levels, in lumbar pathology, the first approach is rehabilitation focused on restoring range of motion and muscular strengthening. Surgery is reserved to symptomatic patients non-responsive to non-operative treatment with imaging evidence of spinal cord compression. Lumbar disc herniation successfully receives non-operative treatment in 90% of patients [93]. Professional athletes who underwent surgery, both total disc replacement or discectomy and fusion, experience significant improvement of symptoms in a high percentage of cases and about 95% of patients return to sport without significant differences with previous sports performances [94, 95]. Lumbar discectomy offers variable results according to the type of sport practiced by professional athletes. Satisfactory outcomes and high rates of return to sport can be found in American football players [96]. On the contrary, baseball players who underwent surgery had a shorter career compared to colleagues with the same diagnosis but treated conservatively [97]. This heterogeneity highlights the importance of load forces as causes of lumbar hernia and possible post-surgical recurrence. Treatment should be guided by surgeon experience and set on athlete’s functional requirements.

### Upper limb

#### Shoulder

In athletes, shoulder injuries commonly involve rotator cuff tendons and the labrum.

##### Superior labrum anterior to posterior (SLAP) lesions

Snyder reported an incidence of SLAP lesions ranging from 4.8 to 6.9% [98, 99]. The injury mechanism is usually overhead activity that determines a contact between the articular side of the supraspinatus tendon and the postero-superior glenoid labrum [98].

The first line of treatment can be conservative, mainly aiming to scapula stabilization, rotator cuff strengthening, and stretching of the postero-inferior capsule. Indeed, an excessive loss of glenohumeral internal rotation (GIRD) is common in overhead athletes [100].

Surgical indication depends on several factors, such as the kind of lesion, the patient’s age, level of activity, and the previous non-operative management. Type II SLAPs are the most common. They present complete detachment of the superior labrum and the biceps anchor from the superior glenoid tubercle. Repairing the lesion in older patients (> 40 years old) showed higher rate of unsatisfactory outcomes and surgical revision. In this instance, the tendon of the long head of the biceps can undergo either a tenodesis or a tenotomy. In young active athletes, the lesion should be repaired [101]. However, this is the most challenging type of lesion: its repair can result in a stiff shoulder [102], and return to play at the pre-injury level cannot be guaranteed [103]. When choosing the treatment method, in type II lesion, it is of paramount to take into account the patient’s type and level of activity. Recent evidences showed that biceps suprapectoral [104] or subpectoral [105] tenodesis is preferable over SLAP repair in young patients practicing overhead activity. Type I lesions are characterized by frying of the labrum, and therefore, the proposed surgical treatment is arthroscopic debridement. Type III lesions show a bucket handle tear of the labrum, while the biceps tendon is normal. For these lesions, resection of the unstable bucket handle tear is indicated [101]. Type IV is a type III lesion with a longitudinal lesion of the biceps tendon, which may dislocate into the joint. When less than 30% of the tendon is torn, the lesion can be abraded together with the degenerated area of the labrum. If the lesion involves more than 30% of the biceps tendon, it will be repaired in young patients or excised in older ones [106].

##### Bankart lesion

Anterior shoulder dislocation is common especially in contact athletes such as American football, ice hockey, and rugby players [107]. The most common injury mechanism in athletes is abduction and external rotation of the arm by an externally rotating force [108]. This injury mechanism generates a Bankart lesion where the antero-inferior capsule-labral complex is detached from the glenoid rim either alone or with a bony fragment (bony Bankart). In athletes, even after the first dislocation, non-operative treatment leads to a high recurrence rate [109].

Arthroscopic repair of the Bankart lesion has become more popular than open surgery, with similar reoperation and recurrence rate [110]. Return to sport after arthroscopic Bankart repair is variable, going up to 95% [111]. In bony Bankart lesions, following the new concept of glenoid track introduced by Itoi in 2007 [112], there has been much debate on which is the best treatment to address both the humeral (Hill-Sachs) and the glenoid bony lesions. It is suggested to perform arthroscopic Bankart repair for patients with glenoid bone loss < 25% and on-track Hill-Sachs (HS). If the HS is off-track, a remplissage procedure is necessary to restore shoulder stability [113, 114]. When facing a glenoid bone loss > 25%, whether it is on-track or off-track HS, the Latarjet procedure is recommended [115].

##### Rotator cuff tears

Throwing athletes are at higher risk not only for labrum injuries but also for rotator cuff tears. In addition to the common cuff lesions, the throwing athletes show a higher incidence of partial-thickness articular-sided tears of the postero-superior portion of the cuff compared to the general population. This is secondary to the internal impingement syndrome, a phenomenon described by Jobe and Walch [116, 117].

That syndrome is due to a conflict between the posterior glenoid rim and the postero-superior rotator cuff insertion on the greater tuberosity. Factors associated with this condition are recurring microtrauma, scapular diskynesis, and posterior capsule contracture with consequent loss of internal rotation [118].

Surgical options are taken into consideration when conservative measures have failed. In general, partial tears are repaired when the tear involves more than 50% of the tendon thickness; otherwise, the debridement alone is enough [119]. However, it is controversial whether to repair partial lesions of the rotator cuff in a throwing athlete because of the difficulties of returning to play after a rotator cuff repair [120–122].

## Conclusions

The careful application of prevention, surgical, and rehabilitation principles allows orthopedic sports surgeon to take good care of elite athletes and return them to successful sports practice. However, there are some pathologies such as FAI, chronic ankle instability, and rotator cuff tear which lack for clear evidences based on high-level studies with regard to the best treatment option. Further investigations are needed, keeping in mind that newer is not always better and that in many instances a skeptical attitude should be maintained towards miracle cures and shining novelties.

## Abbreviations

ACDF: Anterior cervical discectomy and fusion
ACL: Anterior cruciate ligament
ACLR: ACL reconstruction
ATFL: Anterior talo-fibular ligament
BPTB: Bone patellar tendon bone
CFL: Calcaneofibular ligament
ESWT: Extracorporeal shock waves therapy
FAI: Femoroacetabular impingement
GIRD: Loss of glenohumeral internal rotation
HS: Hill-Sachs
MCL: Medial collateral ligament
MRI: Magnetic resonance imaging
OCL: Chondral lesions of the talus
PCL: Posterior cruciate ligament
RICE: Rest, ice [cryotherapy], compression, and elevation
SLAP: Superior labrum anterior to posterior
